# Precise large deviations of aggregate claims in a size-dependent renewal risk model with stopping time claim-number process

**DOI:** 10.1186/s13660-017-1364-5

**Published:** 2017-04-21

**Authors:** Shuo Zhang, Dehui Wang, Shihang Yu

**Affiliations:** 1 Institute of Mathematics, Jilin University, Changchun, 130012 China; 2 School of Science, Qiqihar University, Qiqihar, 161006 China

**Keywords:** 60F10, 91B30, 60K05, aggregate claims, stopping time, dependent renewal risk model, precise large deviations

## Abstract

In this paper, we consider a size-dependent renewal risk model with stopping time claim-number process. In this model, we do not make any assumption on the dependence structure of claim sizes and inter-arrival times. We study large deviations of the aggregate amount of claims. For the subexponential heavy-tailed case, we obtain a precise large-deviation formula; our method substantially relies on a martingale for the structure of our models.

## Introduction

Consider the following renewal risk model. Let $\{X_{k}, k\in \mathbb{N}\}$ and $\{\theta_{k}, k\in \mathbb{N}\}$ be claim sizes and inter-arrival times, respectively. Assume that $(X_{k},\theta_{k})$, $k\in \mathbb{N}$, form a sequence of independent and identically distributed (i.i.d.) copies of a generic random pair $(X,\theta)$ with marginal distribution functions $F=1-\overline{F}$ on $[0,\infty)$ and *G* on $[0,\infty)$, with dependent components *X* and *θ*. The claim arrival times are $\tau_{k}=\sum_{i=1}^{k}\theta_{i}, k\in \mathbb{N}$, with $\tau_{0}=0$. The number of claims is defined by

1.1 $$\begin{aligned} N_{t}^{*}=\inf_{k}\{k\in \mathbb{N}: \tau_{k}\geq t\}, \end{aligned}$$

then $N_{t}^{*}$ is a stopping time. In this way, the aggregate amount of claims over the $[0,t]$ is of the form

1.2 $$\begin{aligned} S_{t}^{*}=\sum_{k=1}^{N_{k}^{*}}X_{k}, \qquad S_{0}^{*}=X_{0}=0,\quad t\geq0. \end{aligned}$$

Note that $N_{t}^{*}=\sup_{k}\{k\in \mathbb{N}:\tau_{k}\leq t\}\triangleq N_{t}$ if $\tau_{k}=t$, whereas $N_{t}^{*}=N_{t}+1$ if $\tau_{k}\neq t$.

We study large deviations of $S_{t}^{*}$ in (1.2). We only consider the case of heavy-tailed claim-size distributions. One of the most important classes of heavy-tailed distributions is the class $\mathcal{S}$ of subexponential distributions. By definition, a distribution *F* on $[0,\infty)$ is subexponential if $\overline{F}(x)=1-F(x)$ for all $x\geq0$ and the relation

1.3 $$\begin{aligned} \lim_{x\rightarrow\infty}\frac{\overline {F^{*n}}(x)}{\overline{F}(x)}=n \end{aligned}$$

holds for all $n\geq2$, where $F^{*n}$ denotes the *n*-fold convolution of *F*. Clearly, (1.3) implies

1.4 $$\begin{aligned} \lim_{x\rightarrow\infty}\frac{P(X_{1}+X_{2}+\cdots +X_{n}>x)}{P(\max\{ X_{1},\ldots,X_{n}\}>x)}=1, \end{aligned}$$

where $X_{1},X_{2},\ldots $ is a sequence of i.i.d. r.v.’s with the common distribution function (d.f.) *F*. See Embrechts *et al.* [1] for a nice review of subexponential distributions in the context of insurance and finance.

Our main result is given now.

### Theorem 1.1

*Consider the aggregate amount of claims* $S_{t}^{*}$ *in* (1.2), *assume that* $F\in\mathcal{S}$, $E[X]=\mu\in(0,\infty)$ *and* $E[\theta]=1/\lambda\in(0,\infty)$. *Then*, *for arbitrarily given* $\gamma>0$, *we have uniformly for all* $x\geq\gamma t$

1.5 $$\begin{aligned} P\bigl(S_{t}^{*}-\mu\lambda t >x\bigr)\thicksim\lambda t \overline{F}(x), \quad t\rightarrow\infty. \end{aligned}$$

A non-standard renewal risk model with dependent components *X* and *θ*, which was firstly proposed by Albrecher and Teugels [2] and further studied by Boudreault *et al.* [3], Cossette *et al.* [4], Badescu *et al.* [5], and among many others. Recently, Asimit and Badescu [6] introduced a general dependence structure for $(X,\theta)$, via the conditional tail probability of *X* given *θ*; see also Li *et al.* [7]. In particular, Chen and Yuen [8] considered that there is a random variable *θ̃* such that

1.6 $$\begin{aligned} \operatorname{Pr}(\theta>t|X>x)\leq \operatorname{Pr}(\tilde{\theta}>t) \end{aligned}$$

holds for $t\geq0$ and large enough *x*, they studied the large deviations of the aggregate amount of $\mathcal{C}$ heavy-tailed claims, where $\mathcal{C}\subset\mathcal{S}$ (see Embrechts *et al.* [1]).

We now comment on the approaches used in this work. First, in Theorem 1.1, we do not make an assumption on the dependence structure of $(X,\theta)$. The existing results usually require a conditional tail probability of *X* given *θ*, *e.g.*, Chen and Yuen [8] made the assumption (1.6), to say the least. Second, we extend the asymptotic behavior of the large deviations of $S_{t}^{*}$ to the case of $\mathcal{S}$ heavy-tailed claims. Finally, we construct a martingale to prove our result.

The rest of the paper is organized as follows. Section 2 recalls various preliminaries and prepares a few lemmas. Section 3 presents the proof of the main result. We end the paper with conclusions in Section 4.

## Preliminaries

Throughout this paper, for two positive functions $a(\cdot)$ and $b(\cdot)$, we write $a(x)\lesssim b(x)$ if $\limsup_{x\rightarrow \infty}a(x)/b(x)\leq1$, write $a(x)\gtrsim b(x)$ if $\liminf_{x\rightarrow\infty}a(x)/b(x)\geq1$, and write $a(x)\sim b(x)$ if both. Very often we equip limit relationships with certain uniformity, which is crucial for our purpose. For instance, for two positive bivariate functions $a(\cdot,\cdot)$ and $b(\cdot,\cdot)$, we say that $a(\cdot,\cdot)\sim b(\cdot,\cdot)$ holds uniformly for $x\in\Delta\neq\emptyset$ if

$$\lim_{t\rightarrow\infty}\sup_{x\in\Delta } \biggl\vert \frac{a(x;t)}{b(x;t)}-1 \biggr\vert =0. $$

Clearly, the asymptotic relation $a(\cdot,\cdot)\sim b(\cdot,\cdot)$ holds uniformly for $x\in\Delta$ if and only if

$$\limsup_{t\rightarrow\infty}\sup_{x\in\Delta } \frac{a(x;t)}{b(x;t)} \leq1\quad \mbox{and}\quad \liminf_{t\rightarrow\infty}\inf _{x\in\Delta } \frac{a(x;t)}{b(x;t)}\geq1. $$

To obtain our desired results, we need to mention the following useful lemma.

### Lemma 2.1

*Consider the renewal counting process* $N_{t}^{*}$ *in* (1.1). *Under the assumption* $E[\theta]=1/\lambda<\infty$. *Then*, *for any* $p\geq1$, *we have*

2.1 $$\begin{aligned} E\bigl[N_{t}^{*}\bigr]^{p}\sim(\lambda t)^{p}, \quad \textit{as }t\rightarrow\infty. \end{aligned}$$

### Proof

First, for arbitrarily $\varepsilon>0$, by definition of $N^{*}$ and $E[\theta]=1/\lambda$, we have

2.2 $$\begin{aligned} \mathbb{P}\bigl\{ N^{*}_{t}>(\lambda+\varepsilon)t\bigr\} =&\mathbb{P}\Biggl\{ \sum_{j=1}^{(\lambda +\varepsilon)t}\theta_{j} \leq t\Biggr\} \\ =&\mathbb{P}\Biggl\{ \frac{1}{(\lambda+\varepsilon)t}\sum_{j=1}^{(\lambda +\varepsilon)t} \theta_{j}\leq\frac{1}{\lambda+\varepsilon}\Biggr\} \\ \rightarrow&0. \end{aligned}$$

Similarly,

2.3 $$\begin{aligned} \mathbb{P}\bigl\{ N^{*}_{t}< (\lambda-\varepsilon)t\bigr\} \rightarrow0. \end{aligned}$$

Combining (2.2) and (2.3), we have

2.4 $$\begin{aligned} \frac{N_{t}^{*}}{t}\stackrel{P}{\rightarrow}\lambda. \end{aligned}$$

It remains to show

$$\begin{aligned} \sup_{t\geq1}\mathbb{E}\biggl(\frac{N_{t}^{*}}{t} \biggr)^{p}< \infty,\quad \mbox{for } \forall p\geq1. \end{aligned}$$

Indeed, for any $\delta>0$,

2.5 $$\begin{aligned} \mathbb{E}\biggl(\frac{N_{t}^{*}}{t} \biggr)^{p} =&p \int_{0}^{\infty}u^{p-1}\mathbb{P}\bigl\{ N_{t}^{*}>ut\bigr\} \, du \\ =&p(\lambda+\delta)^{p} \int_{0}^{\infty}u^{p-1}\mathbb{P}\bigl\{ N_{t}^{*}>(\lambda +\delta )ut\bigr\} \,du \\ \leq&p(\lambda+\delta)^{p} \int_{0}^{\infty}u^{p-1}\mathbb{P}\Biggl\{ \sum _{j=1}^{(\lambda+\delta)ut}\theta_{j}\leq t\Biggr\} \,du. \end{aligned}$$

For the case when $u\geq1$ is an integer

$$\begin{aligned} \mathbb{P}\Biggl\{ \sum_{j=1}^{(\lambda+\delta)ut} \theta_{j}\leq t\Biggr\} \leq&\mathbb{P}\Biggl\{ \bigcap _{k=1}^{u} \Biggl\{ \sum_{j=(k-1)(\lambda+\delta)t+1}^{k(\lambda+\delta)t} \theta_{j}\leq t \Biggr\} \Biggr\} = \Biggl(\mathbb{P}\Biggl\{ \sum_{j=1}^{(\lambda+\delta)t}\theta _{j}\leq t \Biggr\} \Biggr)^{u}. \end{aligned}$$

By the law of large numbers

$$\mathbb{P}\Biggl\{ \sum_{j=1}^{(\lambda+\delta)t} \theta_{j}\leq t \Biggr\} \rightarrow0\quad (t\rightarrow\infty). $$

So we have the bound, for any constant $c>0$,

2.6 $$\begin{aligned} \mathbb{P}\Biggl\{ \sum_{j=1}^{(\lambda+\delta)ut} \theta_{j}\leq t\Biggr\} \leq e^{-cu}. \end{aligned}$$

Combining (2.5) and (2.6), uniformly for large *u* and *t*,

2.7 $$\begin{aligned} \mathbb{E}\biggl(\frac{N_{t}^{*}}{t} \biggr)^{p} \leq&p(\lambda+ \delta)^{p} \int _{0}^{\infty }u^{p-1}e^{-cu}\,du< \infty. \end{aligned}$$

By (2.4) and (2.7), we obtain Lemma 2.1. □

## Proof of Theorem 1.1

By $F\in\mathcal{S}$ and (1.4), we need only to prove

$$\mathbb{P}\Bigl\{ \max_{k\leq N_{t}^{*}}(X_{k}-\mu)>x \Bigr\} \sim \lambda t\overline{F}(x)\quad \mbox{for }t,x\rightarrow\infty. $$

Write $\xi_{k}=I_{\{X_{k}-\mu>x\}}$. First,

$$I_{\{\max_{k\leq N_{t}^{*}}(X_{k}-\mu)>x\}}\leq\sum_{k=1}^{N_{t}^{*}} \xi_{k}. $$

By Wald’s equation

$$\mathbb{P}\Bigl\{ \max_{k\leq N_{t}^{*}}(X_{k}-\mu)>x\Bigr\} \leq\bigl(\mathbb{E}N_{t}^{*}\bigr)\mathbb{P}\{X>x+\mu\}. $$

From Lemma 2.1 and $\overline{F}(x+\mu)\sim\overline{F}(x) $ (see class $\mathcal {S}\subset\mathcal{L}$ and definition of $\mathcal{L}$, Embrechts *et al.* [1]), we have

3.1 $$\begin{aligned} \mathbb{P}\Bigl\{ \max_{k\leq N_{t}^{*}}(X_{k}-\mu)>x \Bigr\} \lesssim \lambda t\overline{F}(x)\quad \mbox{for }t,x\rightarrow\infty. \end{aligned}$$

On the other hand,

3.2 $$\begin{aligned} I_{\{\max_{k\leq N_{t}^{*}}(X_{k}-\mu)>x\}}\geq\sum_{k=1}^{N_{t}^{*}} \xi_{k}-\sum_{1\leq j< k\leq N_{t}^{*}}\xi_{j} \xi_{k}. \end{aligned}$$

All we need is to bound the second term. Notice that

$$M_{n}\equiv\sum_{1\leq j< k\leq n}\xi_{j}( \xi_{k}-\mathbb{E}\xi_{k}),\quad n=1,2,\ldots $$

is a martingale. By Doob’s stopping rule,

$$\mathbb{E}M_{N_{t}^{*}}=\mathbb{E}M_{1}=0. $$

Or

$$\mathbb{E}\biggl( \sum_{1\leq j< k\leq N_{t}^{*}}\xi_{j}( \xi_{k}-\mathbb{E}\xi_{k}) \biggr)=0. $$

Hence,

$$\mathbb{E}\biggl( \sum_{1\leq j< k\leq N_{t}^{*}}\xi_{j} \xi_{k} \biggr)=\mathbb{E}\xi\cdot \mathbb{E}\biggl( \sum_{1\leq j< k\leq N_{t}^{*}} \xi_{j} \biggr)=\mathbb{E}\xi\cdot \mathbb{E}\Biggl( \sum_{j=1}^{ N_{t}^{*}-1} \xi_{j}\bigl(N_{t}^{*}-j\bigr) \Biggr). $$

Write $Z_{n}=\sum_{j=1}^{n}\xi_{j}$. Notice that

$$\sum_{j=1}^{ N_{t}^{*}-1}\xi_{j} \bigl(N_{t}^{*}-j\bigr)=\sum_{n=1}^{N_{t}^{*}-1}Z_{n}. $$

Therefore,

3.3 $$\begin{aligned} \mathbb{E}\Biggl(\sum_{j=1}^{ N_{t}^{*}-1} \xi_{j}\bigl(N_{t}^{*}-j\bigr) \Biggr) =&\mathbb{E}\Biggl(\sum _{n=1}^{N_{t}^{*}-1}Z_{n} \Biggr) \\ =&\sum_{n=1}^{\infty} \mathbb{E}(Z_{n} I_{\{N_{t}^{*}\geq n+1\}}) \\ =&\sum_{n=1}^{\infty} \mathbb{E}(Z_{n} I_{\{\tau_{n+1}\leq t\}}). \end{aligned}$$

For each *n*,

3.4 $$\begin{aligned} \mathbb{E}(Z_{n} I_{\{\tau_{n+1}\leq t\}}) =&\sum _{j=1}^{n}\mathbb{E}(\xi_{j} \cdot I_{\{\tau_{n+1}\leq t\}}) \\ \leq&\sum_{j=1}^{n}\mathbb{E}(\xi_{j} \cdot I_{\{\sum_{k\neq j}^{n+1}\theta_{k}\leq t\} } ) \\ =&\sum_{j=1}^{n}\mathbb{E}\xi \cdot \mathbb{P}\Biggl\{ \sum _{k=1}^{n}\theta_{k}\leq t\Biggr\} =n\mathbb{E}\xi\cdot \mathbb{P}\bigl\{ N_{t}^{*}\geq n\bigr\} . \end{aligned}$$

By (3.3) and (3.4), $\exists c>0$ such that

$$\mathbb{E}\Biggl(\sum_{j=1}^{ N_{t}^{*}-1}\xi_{j} \bigl(N_{t}^{*}-j\bigr) \Biggr)\leq \mathbb{E}\xi\cdot\sum _{n=1}^{\infty}n \mathbb{P}\bigl\{ N_{t}^{*}\geq n\bigr\} \leq c\cdot \mathbb{E}\xi\cdot \mathbb{E}\bigl(N_{t}^{*}\bigr)^{2}. $$

Combining our computation and Lemma 2.1,

$$\mathbb{E}\biggl( \sum_{1\leq j< k\leq N_{t}^{*}}\xi_{j} \xi_{k} \biggr)\leq c\cdot(\mathbb{E}\xi)^{2}\cdot \mathbb{E}\bigl(N_{t}^{*}\bigr)^{2}\leq c\bigl(\lambda t\cdot \overline{F}(x)\bigr)^{2}=o\bigl(\lambda t\overline{F}(x)\bigr). $$

By (3.2), therefore,

3.5 $$\begin{aligned} \mathbb{P}\Bigl\{ \max_{k\leq N_{t}^{*}}(X_{k}-\mu)>x \Bigr\} \gtrsim\lambda t\overline{F}(x)\quad \mbox{for }t,x\rightarrow\infty. \end{aligned}$$

Hence, by (3.1) and (3.5), we have

$$\mathbb{P}\Bigl\{ \max_{k\leq N_{t}^{*}}(X_{k}-\mu)>x \Bigr\} \sim \lambda t\overline{F}(x)\quad \mbox{for }t,x\rightarrow\infty. $$

## Conclusions

As was remarked by a few researchers in the area, precise large-deviation results of size-dependent renewal risk models are particularly useful for evaluating some risk measures such as the conditional tail expectation of the aggregate amount of claims from a large insurance portfolio. Finally, we would like to point out that equation (1.5) agrees with existing ones in the literature. This indicates that the aggregate amount of $S_{t}^{*}$ defined by (1.1) does not affect the asymptotic behavior of the large deviations.

## References

[1] Embrechts P, Kltippelberg C, Mikoseh T (1997). Modeling Extremal Events for Insurance and Finance.

[2] Albrecher H, Teugels JL (2006). Exponential behavior in the presence of dependence in risk theory. J. Appl. Probab..

[3] Boudreault M, Cossette H, Landriault D, Marceau E (2006). On a risk model with dependence between interclaim arrivals and claim sizes. Scand. Actuar. J..

[4] Cossette H, Marceau E, Marri F (2008). On the compound Poisson risk model with dependence based on a generalized Farlie-Gumbel-Morgenstern copula. Insur. Math. Econ..

[5] Badescu AL, Cheung ECK, Landriault D (2009). Dependent risk models with bivariate phase-type distributions. J. Appl. Probab..

[6] Asimit AV, Badescu AL (2010). Extremes on the discounted aggregate claims in a time dependent risk model. Scand. Actuar. J..

[7] Li J, Tang Q, Wu R (2010). Subexponential tails of discounted aggregate claims in a time-dependent renewal risk model. Adv. Appl. Probab..

[8] Chen Y, Yuen KC (2012). Precise large deviations of aggregate laims in a size-dependent renewal risk model. Insur. Math. Econ..

